# How new genes are born

**DOI:** 10.7554/eLife.55136

**Published:** 2020-02-19

**Authors:** Urminder Singh, Eve Syrkin Wurtele

**Affiliations:** Department of Genetics, Developmental and Cell Biology, Iowa State UniversityAmesUnited States

**Keywords:** orphan genes, genetic novelty, sequence divergence, evolution, conserved synteny, de novo gene emergence, *D. melanogaster*, Human, *S. cerevisiae*

## Abstract

Analysis of yeast, fly and human genomes suggests that sequence divergence is not the main source of orphan genes.

**Related research article** Vakirlis N, Carvunis A-R, McLysaght A. 2020. Synteny-based analyses indicate that sequence divergence is not the main source of orphan genes. *eLife* **9**:e53500. doi: 10.7554/eLife.53500

For half a century, most scientists believed that new protein-coding genes arise as a result of mutations in existing protein-coding genes. It was considered impossible for anything as complex as a functional new protein to arise from scratch. However, every species has certain genes, known as 'orphan genes', which code for proteins that are not homologous to proteins found in any other species. What do these orphan genes do, and how are they formed?

To date the roles of hundreds of orphan genes have been characterized. Although this is just a tiny fraction of the total, it is known that most of them code for proteins that bind to conserved proteins such as transcription factors or receptors. Some of these proteins are toxins, some are involved in reproduction, some integrate into existing metabolic and regulatory networks, and some confer resistance to stress (Carvunis et al., 2012; Li et al., 2009; Xiao et al., 2009; Arendsee et al., 2014; Belcaid et al., 2019). However, none of them are enzymes (Arendsee et al., 2014). Orphan genes arise quickly, so they may provide a disruptive mechanism that allows a given species to survive changes to its environment. Thus, the study of how orphan genes arise (and fall) is central to understanding the forces that drive evolution (Figure 1).

**Figure 1.**
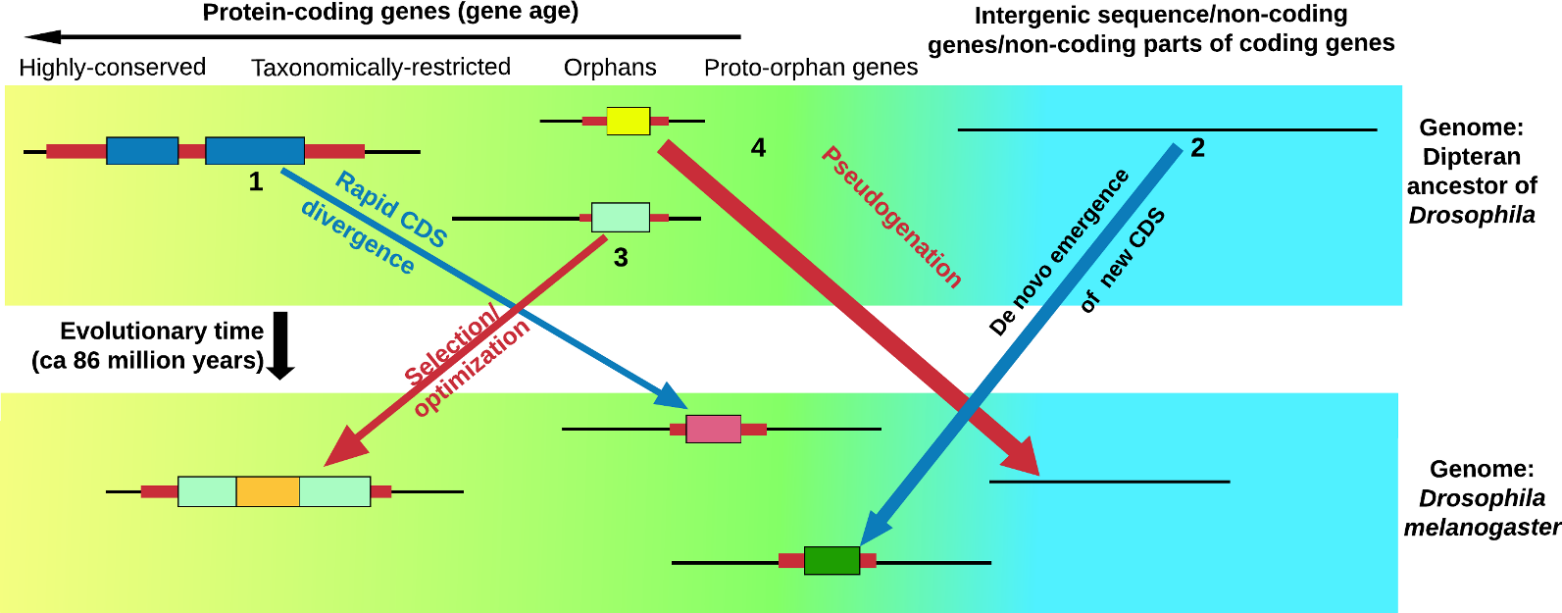
Life cycle of orphan genes. Every species has orphan genes that have no homologs in other species. This schematic shows the genome of the fruit fly (bottom) and the genome of an ancestor of the fruit fly (top). Each panel also shows (from left to right): genes that are highly conserved and can be traced back to prokaryotic organisms (yellow background); genes that are found in just a few related species (taxonomically restricted genes), orphan genes and potential orphan genes that are not currently expressed and are thus free from selection pressure (proto-orphan genes); and regions of the genome that do not code for proteins (blue background) (Van Oss and Carvunis, 2019; Palmieri et al., 2014). An orphan gene can form through the rapid divergence of the coding sequence (CDS) of an existing gene (1), or arise de novo from regions of the genome that do not code for proteins (including the non-coding parts of genes that evolve to code for proteins; 2). Some orphan genes will be important for survival, and will thus be selected for and gradually optimized (3). This means that the genes in a single organism will have a gradient of ages (Tautz and Domazet-Lošo, 2011). Many proto-orphan genes will undergo pseudogenation (that is, they will not be retained; 4). Coding sequences (shown as thick colored bars) with detectable homology are shown in similar colors. Vakirlis et al. estimate that a minority of orphan genes have arisen by divergence of the coding sequence of existing genes.

One possible mechanism is the *'*de novo' appearance of a gene from an intergenic region or a completely new reading frame within an existing gene (Tautz and Domazet-Lošo, 2011). An alternative mechanism is that the coding sequence of the orphan gene arises by rapid divergence from the coding sequence of a preexisting gene: this would mean that an entire set of regulatory and structural elements would be available to the gene as it evolves. Now, in eLife, Nikolaos Vakirlis and Aoife McLysaght (both from Trinity College Dublin) and Anne-Ruxandra Carvunis (University of Pittsburgh) report how they have studied yeast, fly and human genes to compare the contributions of these two mechanisms to the emergence of orphan genes (Vakirlis et al., 2020).

Previous studies have used simulations to estimate the number of orphan genes that appear by divergence; until now, no one had relied on actual genomics data to study this phenomenon. Vakirlis et al. use a new approach to analyze orphan genes that have originated through divergence. They examine regions of the genome that correspond to each other (so-called syntenic regions) in related species to determine whether a gene exists in both regions and, if so, whether the proteins are non-homologous. If the genes have no homology, they may have originated by rapid divergence from the coding sequence of a preexisting gene.

Using this method, Vakirlis et al. infer that at most 45% of *S. cerevisiae* (yeast) orphan genes, 25% of *D. melanogaster* (fruit fly) orphan genes, and 18% of human orphan genes arose by rapid divergence, but this is an upper estimate. For example, it is possible that a new coding sequence might have arisen de novo within an existing gene, rather than the existing coding sequence having been modified beyond recognition.

But how can a protein sequence continue to be selected for as it rapidly diverges? Vakirlis et al. suggest that divergence might occur by a process of partial pseudogenation: the existing gene becomes non-functional, and then, with no selection pressure to retain the old protein, it diverges to form an orphan gene.

Many orphan genes may not have been identified yet, because they do not have homologs in other species, and have few recognizable sequence features. This means that up to 80% of orphan genes can be missed when a new genome is annotated (Seetharam et al., 2019). The approach detailed by Vakirlis, Carvunis and McLysaght evaluates specifically those annotated orphan genes for which a similar gene exists in a related species (which is ~50% of them; Arendsee et al., 2019). As high-quality genomes from more species become available, and as more orphan genes are annotated, the approach will provide yet deeper insights into the origin of these genes.

One of the many open questions in this field deals with genes of ‘mixed age’. Some such genes have incorporated ‘chunks’ of orphans into their coding sequences. A gene that has done this is (somewhat arbitrarily) considered to be the age of its most ancient segment, but we know little about the mechanism of this process or its significance. Another question involves the unique strategies and rates of evolution of each gene (Revell et al., 2018). How might the abundance and mechanisms of orphan gene origin vary among species? And how do different environments affect the emergence of orphan genes?
